# Recoverable resources from pot ale & spent wash from Scotch Whisky production

**DOI:** 10.1016/j.resconrec.2021.106114

**Published:** 2022-04

**Authors:** Christine Edwards, Calum C. McNerney, Linda A. Lawton, Joseph Palmer, Kenneth Macgregor, Frances Jack, Peter Cockburn, Amy Plummer, Alison Lovegrove, Abigail Wood

**Affiliations:** a The School of Pharmacy and Life Science, Robert Gordon University, Aberdeen, AB10 7GJ, United Kingdom; b The Scotch Whisky Research Institute, The Robertson Trust Building, Research Avenue North, Riccarton, Edinburgh, EH14 4AP, United Kingdom; c Diageo, International Technical Centre, Glenochil, Menstrie, Clackmannanshire, Scotland, FK11 7ES, United Kingdom; d Rothamsted Research, West Common, Harpenden, Hertfordshire, AL5 2JQ, United Kingdom

**Keywords:** Pot ale, Resource recovery, Lactic acid, Magnesium, Critical raw materials, Phosphate

## Abstract

Scotch Whisky is an important global commodity which generates extensive co-product known as pot ale or spent wash (> 10 L co-product per L whisky). Whilst this is often used as fertiliser or animal feed, a proportion requires disposal resulting in cost to the distillery along with the negative impact on the carbon footprint due to transportation. This study examined the composition of the soluble fraction of pot ale from twenty-two distilleries in Scotland in order to assess the potential for resource recovery and transition to a more circular economy.

The results reinforced previous studies, demonstrating that pot ale is an excellent source of protein with a potential for recovery >150, 000 t per annum in Scotland based on Whisky production data. Lactic acid, an important industrial platform chemical, was the major organic acid produced with concentrations ranging from 0.3 to 6.6 g *L*^−1^, representing a potential opportunity for recovery for applications such as manufacture of biodegradable polylactic acid for plastics (> 15,000 t per annum based on mean values). Other important platform chemicals, succinic acid and lysine were also identified and considered in sufficient amounts for future use. Pot ale was also shown to contain significant amounts of critical raw materials, magnesium and phosphate, which could be reclaimed for use in fertiliser/feed supporting the development of a new circular economy whilst at the same time reducing the burden of mining and transportation on the environment. The data in this study demonstrated a potential 13.8 kt recoverable phosphate per annum representing more than half of the annual fertiliser consumption in Scotland.

Whisky co-products can contribute to sustainable energy, food and platform chemicals with the added value that metal concentrations are not sufficiently high to prevent its utilisation.

## Highlights

• Detailed characterisation of pot ale and spent wash from 22 Scotch Whisky distilleries.
• Identification of pot ale/spent wash as a renewable source of critical raw materials (including magnesium and phosphate).
• Sustainable resources (protein, carbohydrate, lactic acid, succinic acid, lysine) available in both pot ale and spent wash.
• PCA identified differences in chemical composition between pot ale from malt and spent wash from grain distilleries.

## Graphical abstract

**Figure:**
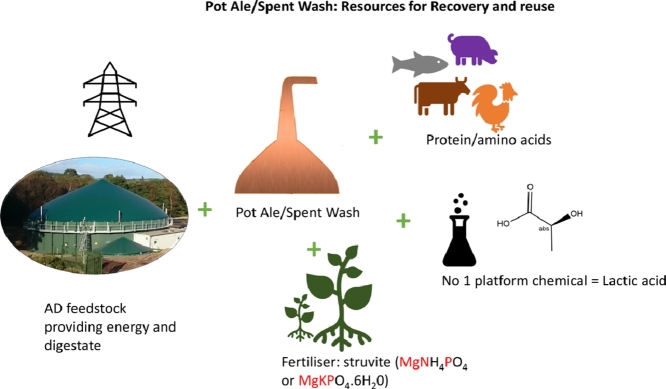

## 1 Introduction

With a global human population of almost 8 billion there is growing pressure on natural resources which impact food, energy and water. Consequently, there has been a paradigm shift towards the development of sustainable circular economies ensuring recovery and reuse to minimise the impact on the environment. It is therefore essential to explore manufacturing co-product streams, understanding composition, stability and seasonality. With this knowledge it is then possible to explore available technologies for resource recovery to achieve sustainability.

The Scotch Whisky industry contributes £5.5 billion per annum to the UK economy and employs 42,000 people, including 10,500 directly in Scotland (SWA, 2019). The production of malt and grain whisky in 2017 exceeded 550 million litres of pure alcohol (mLPA) (Gray, 2018). For every litre of malt whisky produced, approximately 10 L of residue, known as pot ale, remains after the distillation process (Scottish Government, 2015). Similarly, for the production of grain whisky, 18 L of a comparable residue, spent wash, also remains (Scottish Government, 2015). Using annual spirit production data reported by Gray (2018), a total of 7.65 million t of pot ale and spent wash is produced every year in Scotland. This co-product is nutritious with high chemical oxygen demand (COD, 30–50 g/L) and biological oxygen demand (BOD). It is highly aqueous with an insoluble fraction containing yeast, bacteria and grain particles (approx. 30% dry matter) and a soluble fraction containing proteins and a complex mixture of organic and inorganic molecules (approx. 70% dry matter).

In line with the Scotch Whisky Association's environmental strategy to reduce carbon footprint many of these co-products are reused. A significant portion of this is converted into pot ale syrup (PAS) or distillers’ dark grains (PAS combined with draff) and used as animal feed. Although this process is time consuming and energy demanding, the 10-fold reduction in volume decreases the number of tankers required for transportation off-site. In addition to producing animal feed, these co-products are also used as a fertiliser. A small number of distilleries may have permission to dispose of their co-product to sea, where its discharge and subsequent dilution is regulated by the Scottish Environment Protection Agency.

Over the last decade pot ale/spent wash often combined with draff serves as an excellent feedstock for anaerobic digestion (AD) which can provide as much as 50% of the energy requirements necessary to run distillery processes, reducing carbon footprint, environmental impact and dependency on fossil fuels (Diageo, 2015). A distillery producing ∼2.6 mLPA pa would produce ∼6400 t draff and ∼21,000 t of pot ale which could provide ∼7500 MWh of biogas (Personal communication). However, in some locations using co-products for AD feedstock has resulted in a rise in cost or reduced availability of the same co-products for livestock feed causing some concern for this sector. In addition, digestate, a co-product of AD, is an excellent fertiliser, with improved pH and nutrient availability compared to spreading acidic pot ale on land.

As the Industrial Biotechnology sector in Scotland is expected to grow to approximately £900 m by 2025 (Scottish Enterprise, 2019), emerging Small, medium enterprises (SMEs) hope to utilise pot ale and spent wash to extract higher value chemicals either to replace or complement current distillery practices. Several studies have already demonstrated potential approaches for valorisation of whisky co-products including a novel process to recover protein from pot ale providing a sustainable, environmentally friendly, high quality aquaculture feedstock (Traub Modinger, 2015). Commercialisation and scale up of this technology at distillery sites is in progress and the protein has been approved by the EU for use in feed.

The thermal hydrolysis and fermentation of distillery co-product will produce bio-based solvents such as butanol with a much lower carbon footprint when compared to oil-based solvents. A new biorefinery in Grangemouth, Scotland, is due to be commissioned in 2021 where it can process up to 50,000 t pa of distillery co-product. However, it will not be practical to transport pot ale from remote locations such as Islay, (approximately 118, 000 t pa). Ultimately there is a need for information and a valorisation toolbox offering the best solutions to support local communities, agriculture and aquaculture.

Relatively few studies have explored the composition of pot ale. Pot ale from a single distillery has previously been shown to contain copper (2–6 mg *L*^−1^) and high but variable concentrations of volatile organic acids (4–10 g *L*^−1^; Graham et al., 2012). Recent studies exploring the composition of pot ale from a small number of distilleries (Barrena et al., 2018; White et al., 2020) have highlighted the potential of resource recovery (polyphenols, potassium, magnesium and phosphate) in addition to protein recovery. Whilst these studies have highlighted the potential resources available in pot ale/spent wash, it is essential to have an improved understanding of these resources across the industry in order to drive valorisation and circular economy strategies.

This study examines pot ale and spent wash collected from 22 distilleries that were representative, at the time of the study, of the 128 distilleries in production in Scotland. They were spatially distributed across Scotland, encompassing the various whisky regions and covered a range of different Scotch Whisky production parameters. The primary aim of this study was to: (i) quantify concentrations of organic acids, inorganic anions, total organic carbon, metals, protein, amino acids and carbohydrates in pot ale and spent wash; (ii) assess variability in chemical composition; (iii) determine the production factors that may influence the components of pot ale and spent wash; and (iv) review the circular economy opportunities for resource recovery from the soluble fraction of pot ale and spent wash.

## 2 Materials and methods

### 2.1 Distillery selection and the collection of pot ale and spent wash

Pot ale samples, produced from malted barley using a batch distillation process, were collected from twenty malt whisky distilleries in Scotland. A further two spent wash samples were also collected from grain whisky distilleries. The production of grain whisky differs from that of malt whisky, being produced from a mix of un-malted cereals and malted barley through continuous stills. For ease of nomenclature, we refer to these samples as pot ale in the remainder of this paper. Data was collected on the key parameters that might influence the composition of the pot ale, namely barley variety, malt peating level, wort clarity, yeast strain, length of fermentation and time in transit. The distilleries were assigned unique identifier codes in order to keep all disclosed information anonymous.

Distilleries received a sampling kit which included sterile syringes, filters and sterile centrifuge tubes along with the sampling protocol and process questionnaire. Pot ale lines were purged before collection. The pot ale was collected into a clean plastic bucket where a 250–500 mL subsample was taken and thoroughly mixed. Aliquots (3 × 12–14 ml) were filtered through a 0.2 µm MediaKap Plus®hollow fibre filters (Repligen, Netherlands) using a 20 mL syringe and collected into 15 mL sterile centrifuge tubes (Fisher Scientific, UK). Unfiltered pot ale (3 × 15 mL) was also collected in 15 mL tubes for total metal analysis. Filtration was an important practical means to improve sample stability and sterility during transit. Upon receipt samples were frozen and stored at −21 °C prior to analysis.

### 2.2 Compositional analysis of the pot ale samples

#### 2.2.1 Determination of organic acids and inorganic anions

Concentrations of lactate, acetate, phosphate, succinate, nitrate, nitrite, chloride and sulphate were determined in pot ale by Ion Chromatography (IC) using a Dionex Integrion HPIC system (Thermo Fisher Scientific, UK). Analytes were separated on a Dionex IonPac AS11-HC-4 µm column (4 × 250 mm) equipped with a Dionex IonPac AG11-HC-4 µm guard column (4 × 50 mm) and Dionex AERS500 suppressor. A diluted KOH / ultrapure water mobile phase was used with a gradient elution program of the following: 0–8 min (1.5 mM), 8–18 min (15 mM), 18–23 min (15 mM), 23–24 min (24 mM) and 24–30 min (60 mM). A flow rate of 0.38 mL min^–1^ was used. A sample volume of 2 µL was injected once the background conductivity was < 1 µS. Data acquisition and analysis was performed using Chromeleon 7.1 software. Calibration curves were generated using analytical standards (Sigma-Aldrich, UK), at a range of 5–100 mg/L. Pot ale samples were diluted 1/100 prior to analysis. Pot ale samples (triplicate) spiked at a concentration of 10 mg *L*^−1^ using analytical standards of acetate, lactate, chloride, nitrite, succinate, carbonate, sulphate, phosphate and nitrate (Sigma-Aldrich, UK) gave recoveries ranging between 95–103%.

#### 2.2.2 Determination of metals

##### 2.2.2.1 Microwave-assisted acid digestion for total metal content

Pot ale samples were analysed for total metal content in triplicate using an Ethos Easy microwave digestion system (Milestone, UK). Unfiltered pot ale samples were vortexed for 2 min and a 1 mL subsample was added to the digest vessel along with 10 mL of aqua regia (3:1 v/v c.HCl:cHNO_3_; VWR international Ltd, UK). Samples were digested at 200 °C for 90 min, cooled to room temperature before dilution with 15 mL of ultrapure water prior to analysis.

##### 2.2.2.2 Total and soluble metal analysis

Concentrations of Cd (228.802 nm), Cr (267.716 nm), Cu (327.393 nm), Fe (238.204 nm), Ni (231.604 nm), Mg (285.213 nm), Mn (257.610 nm), Pb (220.353 nm) and Zn (206.200 nm) in pot ale and pot ale digests were determined by inductively coupled plasma optical emission spectroscopy (ICP–OES) using a PerkinElmer Optima 8000 DV instrument coupled to a S10 PerkinElmer autosampler (PerkinElmer, UK). Using individual 10,000 mg *L*^−1^ stock solutions (Fisher Scientific, UK) and ultrapure water, calibration standards were prepared at a concentration range of 0.1 to 10 mg *L*^−1^ in triplicate. The argon flow rates were; plasma 15 L min^−1^, auxiliary 0.2 L min^−1^ and nebulizer 0.8 L min^−1^. The ICP-OES was re-calibrated and blanked using ultrapure water prior to each batch of analysis. Samples were injected at a flow rate of 1.5 mL min^−1^. Calibration curves obtained for all elemental analysis were ≥ 0.995 with% RSDs of < 5%.

#### 2.2.3 Total organic carbon analysis

The total organic carbon (TOC) content was determined by wet chemical combustion using a Shimadzu TOC-VCPH total organic carbon analyser connected to a Shimadzu ASV-V autosampler (Shimadzu, UK). The furnace temperature was set at 750 °C with an oxygen carrier gas at a flow rate of 150 mL min^−1^. A total of 25 µL of sample was injected and hydrolysed with phosphoric acid (25% w/w). Samples were analysed in triplicate. The instrument was calibrated using potassium hydrogen phthalate (Sigma-Aldrich, UK) and calibration curves ranged from 100 mg *L*^−1^ to 1000 mg *L*^−1^.

#### 2.2.4 Carbohydrate analysis

##### 2.2.4.3 Determination of total carbohydrates based on glucose response

Using 0.45 μm polyvinylidene fluoride (PVDF) filters (Merck, UK), 1 mL aliquots of pot ale were filtered, freeze-dried and re-suspended to give a concentration of 5 mg mL^−1^ with water. Colorimetric analysis of total carbohydrate was as described by Dubois et al. (1956). Briefly, 20 µL of re-suspended pot ale (equivalent to 0.1 mg) was diluted to 1 mL in water and 25 µL of 80% phenol solution (Fisher Scientific, UK) was added, along with 2.5 mL of concentrated sulfuric acid (Fisher Scientific, UK). Samples were capped, vortexed and left to stand for 10 min, cooled to room temperature prior to reading absorbence at 485 nm. Calibration curves were prepared using glucose at a range of concentrations and quantification was achieved using R^2^ coefficients greater than 0.998. Glucose standards (0.1 mg mL^−1^) were also included with each batch of samples.

##### 2.2.4.4 Determination of free carbohydrates

For the analysis of free monosaccharides (arabinose, rhamnose, galactose, glucose and xylose), aliquots of resuspended pot ale were filtered through 0.45 μm PVDF filters and diluted to 0.15 mg mL^−1^ using ultrapure water. Samples were analysed by high-performance anion-exchange chromatography with pulsed amperometric detector (HPAEC-PAD) using a Dionex ICS-5000+ chromatography system (Thermo Scientific, UK) at a column oven temperature of 30 °C. The analytical column used was a CarboPac PA20 (3 × 150 mm) with CarboPac PA20 guard column (3 × 30 mm). A KOH mobile phase was used at a flow of 0.5 mL min^−1^. The gradient elution programme was as follows; 0–14.5 min (4 mM KOH), 14.5–15.0 min (linear increase to 100 mM KOH), 15–18 min (hold at 100 mM KOH), 18–18.5 min (linear decrease to 4 mM KOH) and 18.5–23.5 min (hold at 4 mM KOH). Data was analysed using Chromeleon 7.2. Calibration curves gave R^2^ coefficients greater than 0.998 and analysis displayed good reproducibility (<0.1 RSD) for all calibration drift standards (*n* = 7).

##### 2.2.4.5 Determination of hydrolysed carbohydrates

Monosaccharides (arabinose, galactose, glucose, mannose and xylose) were determined following acid hydrolysis, as described in Freeman et al. (2017). Briefly, freeze-dried pot ale samples were re-suspended to a concentration of 5 mg mL^−1^ with water. Aliquots equivalent to 1 mg of pot ale were dried by vacuum centrifuge. Aliquots (400 µL) of 2 M trifluoracetic acid (Merck, UK), were added to the samples and then incubated for 1 h at 120 °C and left to cool in ice. Hydrolysed samples were again dried by vacuum centrifuge, washed with 500 µl of water and dried again (to remove residual trifluoracetic acid). The samples were re-suspended in 1 mL of ultrapure water, centrifuged at 13,400x g for 2 min and the supernatant filtered through 0.45 μm PVDF filters. A set of monosaccharides standard sugars were treated in exactly the same manner and analysed alongside a set of standards by HPAEC-PAD as described in section 2.2.4.2.

#### 2.2.5 Analysis of soluble protein

Briefly, pot ale samples were thawed, vortexed, and 50 µl of sample was added to a 1.5 ml Eppendorf tube with 950 µl of 1 M NaOH. Samples were then vortexed for 2 min at 2500 rpm, before heating at 60 °C on a hotplate for 10 mins. Samples were vortexed at 14,000x g for 5 min to remove solids. Five-hundred microlitres of the supernatant were mixed with 500 µl of freshly-prepared 0.1% CuSO4•5H_2_O solution in 15% NaOH, and absorbence was read on a Biochrom WPA spectrophotometer (Biochrom Ltd, UK) at 310 nm after blanking the spectrophotometer with 1 M NaOH. A standard curve of bovine serum albumin (BSA) was prepared in 1 M NaOH (0 −1500 µg ml^−1^) to quantify soluble protein samples. Spectrophotometer readings were baseline corrected by deducting absorbence at 800 nm to account for difference between spectra. Seven-blank samples (1 M NaOH) were also analysed.

#### 2.2.6 Amino acid analysis

Mixed calibrations standards (0.1—–5.0 mg *L*^−1^) were prepared from 21 individual amino acids (alanine, arginine, asparagine, aspartic acid, cysteine, glutamic acid, glutamine, glycine, histidine, hydroxyproline, isoleucine, leucine, lysine, methionine, phenylalanine, proline, serine, threonine, tryptophan, tyrosine and valine; Sigma-Aldrich, UK) with o-Methyl Tyrosine (1.0 mg *L*^−1^) used as an internal standard. Quantification was based on calibration curves with R^2^ coefficients of > 0.990. Samples were diluted in a 5% ethanol solution prior to analysis. Standards and samples were derivatised prior to analysis using an AccQTag™ derivatisation kit (Waters, UK). Quantification was carried out by Liquid Chromatography-Mass Spectrometry (LC-MS) employing a Waters Acquity UPLC coupled to an Agilent (UK) 6150B single quad MS on single ion monitoring detection mode. Separation of derivatised amino acids was achieved using a Waters Acquity T3, (2.1 × 150 mm long, 1.6 µm particle size) column at a flow rate of 0.5 mL min^−1^ and an injection volume of 0.5 µL. Mobile phases consisting of 0.4% formic acid in water (A) and 0.4% formic acid in acetonitrile (B) were used in a 20 min gradient. The gradient was as follows; 0–1 min (100% A), 1–20 min (99% A), 20–22 min (80% A), 22–25 min (0% A) and 25 min (99% A). The mass spectrometer employed an Agilent Jet Stream ESI ion source using a drying gas flow of 12 L min^–1^, nebuliser pressure of 50 PSI and sheath gas flow of 10 L min^–1^. Both drying gas and sheath gas temperature were set to 350 °C. Capillary and nozzle voltages were 1800 V and 2000 V, respectively. For quality control, a laboratory prepared pot ale was spiked with known concentrations of each amino acid standard. The laboratory pot ale (*n* = 12) gave typical amino acid accuracy and precision within 10% of the target concentration and an RSD ranged from 3.77% (tryptophan) to 19.8% (hydroxyproline). Spiked pot ale recoveries ranged from 83.5% (cysteine) to 119% (arginine). All samples were analysed in triplicate and results above the limit of detection.

#### 2.2.7 Statistical analysis of the compositional data

Principal Component Analysis (PCA) was used to summarise the compositional data. In this method, derived variables known as principal components (PCs) were constructed in order to express a large proportion of the total variance of the original multivariate data with a smaller number of variables. By plotting the PCs, interrelationships between the compositional variables could be viewed along with sample (distillery) patterns, groupings, similarities or differences. This allowed relationships between the process parameters and composition to be explored. The analysis was carried out using Unistat 10 for Excel.

## 3 Results and discussion

### 3.1 Soluble protein

**Fig. 1:**
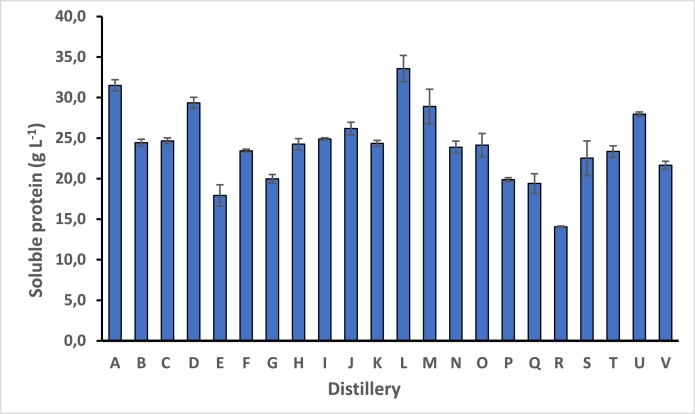
Concentration of soluble protein found in pot ale from each distillery using the micro-biuret method.

Mean concentration of soluble protein content across the distilleries was 20 g *L*^−1^ and ranged from 16–33 g *L*^−^  (Fig. 1) in agreement with past studies and demonstrated the potential of pot ale as a valuable source of food-quality protein for use in animal and aquaculture feed (White et al., 2020). Based on published figures of produced pot ale/spent wash in Scotland, this survey suggests that there is a potential >150, 000 t pa of protein which could be recovered to replace imported soya based protein which was estimated at 102,000 t pa in 2017 (Defra, 2018). Many processes for sustainable protein recovery at scale are currently available including ‘green’ methods which would be very compatible in a biorefinery context, namely isoelectric precipitation or ultrafiltration based techniques (Balandrán-Quintana, 2018).

### 3.2 Free amino acids in pot ale

**Fig. 2:**
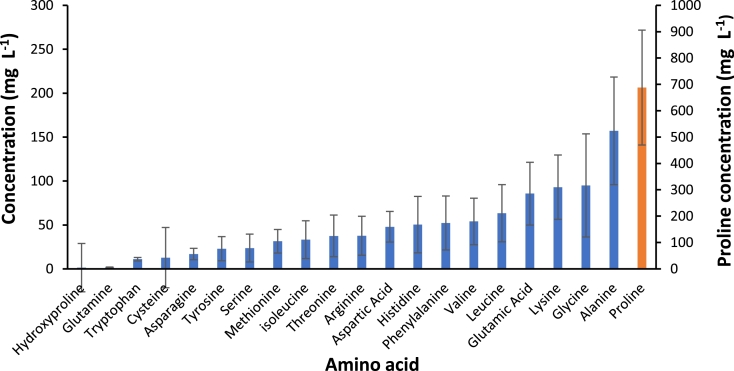
Mean concentration of free amino acids across all distilleries. The error bars are representative of the standard deviation from the mean. The concentration of proline is displayed on the secondary y axis (*n* = 22).

Concentrations of amino acids were generally low as shown in Table S.3, supplementary information. Proline was the most abundant amino acid present in all samples with a mean concentration of 688 mg *L*^−1^ and was significantly higher than the other amino acids detected (Fig. 2). The total amino acid concentrations ranged from 195 to 2737 mg *L*^−1^ with a mean value of 1626 mg *L*^−1^. Amino acids have been produced on an industrial scale by microbial fermentation for more than 50 years. Essential amino acids such as lysine and methionine form a large proportion of the market and are commonly used to fortify the nutritional properties of food and animal feed. Pot ale from distilleries B and I contained >200 mg *L*^−1^ lysine which would represent >20 t lysine for recovery at each distillery which could be used as a high value feed additive. Amino acids such as glutamine are also used to produce flavour enhancers in the food industry (Friedman and Brandon, 2001; Friedman and Finot, 1990; Leuchtenberger et al., 2005). There could be potential for valorisation where significant concentrations of essential amino acids are identified in pot ale.

### 3.3 Organic acids in pot ale

**Fig. 3:**
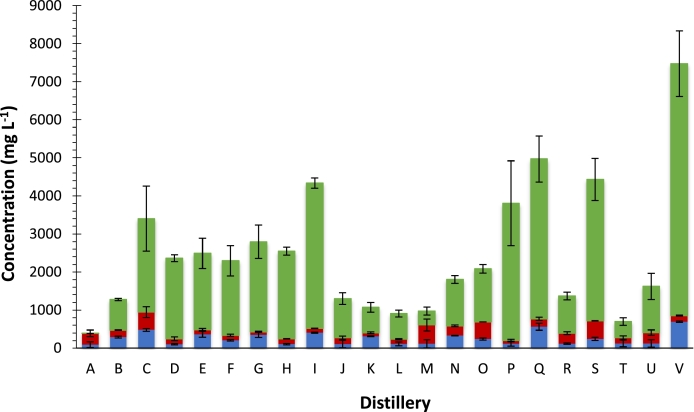
The concentration of organic acids found in pot ale from each distillery. The error bars are representative of the standard deviation of the mean (*n* = 3). Lactic acid (), Acetic acid () and Succinic acid ().

Pot ale/spent wash were shown to contain important platform chemicals lactic, succinic and acetic acid. With the exception of Distillery M, lactic acid was the most abundant organic acid present in pot ale samples analysed. The mean lactic acid concentration was 2 g *L*^−1^ and ranged from 0.36 to 6.6 g *L*^−1^ (Fig. 3). In contrast, mean concentrations of succinic acid (0.26 g *L*^−1^) and acetic acid (0.24 g *L*^−1^) were all approximately an order of magnitude lower. Lactic and acetic acid values in our study agreed with those previously reported (van Beek and Priest, 2002; Tokuda et al., 1999).

The presence of lactic acid is attributed to the activity of lactic acid bacteria during whisky production (Juste et al., 2014: van Beek and Priest, 2002). Lactobacillus strains can survive the mashing process, where the maximum temperature is typically 70 °C (Priest and Pleasants, 1988; Priest, 2004). They typically begin flourishing after the yeast reach stationary phase and will grow on autolysing yeast cells and residual nutrients. The growth of Lactobacilli is encouraged by some distillers to contribute to unique flavour (Priest, 2004; van Beek and Priest, 2002).

Lactic acid is a valuable industrial chemical and was identified as the most important bio-based chemical in a report by The Lignocellulosic Biorefinery Network (LBNet, 2017). It is commonly used as a food additive, as a platform chemical in pharmaceutical products and a wide range of resins and polymers. In addition, it is a key component to produce biodegradable polymer, polylactic acid (PLA). The production of PLA is essential to manufacture a wide range of products such as surgical sutures, disposable plastic packaging and drug delivery systems (Melnicki et al., 2013). In 2013, the estimated global market for lactic acid was 725,000 t pa and around half of this demand was attributed to the bioplastics industry. The bioplastic market is projected to grow by 20% annually due to the increased demand for biodegradable plastic products (Ou et al., 2016; Madhaven-Nampoothiri et al., 2010). Currently, lactic acid can be produced synthetically or through microbial fermentation, although the latter accounts for 90% global production where the preferred single l- or D lactic acid isomer can be obtained with appropriate strain selection. A variety of carbon sources such as pure sugars; lactose, sucrose, or from starch-based materials largely derived from arable crops such as cassava, wheat, corn and barley. Major operational costs include feedstock, pre-treatment, fermentation and downstream processing (Juturu and Wu, 2016). These first-generation feedstocks are of concern as they are in direct competition with food and animal feed markets which questions the sustainability of feedstock available for the production of PLA, particularly due to the increasing global population (Graham-Rowe, 2011). Therefore, there has been increasing interest in using industrial co-products or crop residues. Typical yields of lactic acid fermentative production techniques can be in excess of 100 g *L*^−1^ (Cubas-Cano et al., 2018). Whilst concentrations of lactic acid in this study were lower, recovery from pot ale would have the benefit of being an entirely downstream process resulting in significantly lower operational costs than traditional fermentative production.

### 3.4 Inorganic anions present in pot ale

**Fig. 4:**
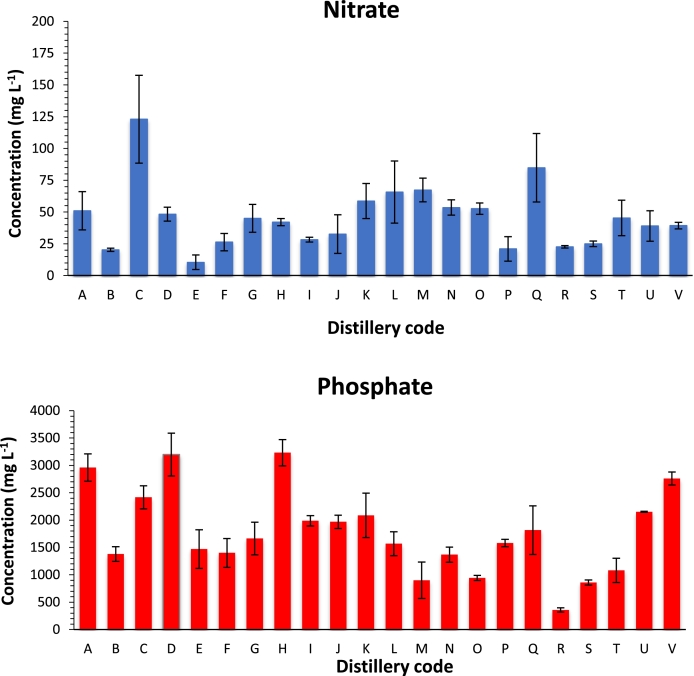
The mean concentration of nitrate and phosphate found in the pot ale samples from each distillery. The error bars are representative of the standard deviation of the mean (*n* = 3).

The key finding for this analysis was that pot ale and spent wash was typically an excellent source of soluble phosphate with concentrations ranging between 0.36–3.2 g *L*^−1^ with mean values of 1.80 g *L*^−1^ (Fig. 4). Phosphorus is a non-renewable resource, essential for the building blocks of RNA, DNA, and membranes along with energy supply (ATP and ADP) of life. Since deposits are finite (400–500 years predicted remaining supply) and there is no alternative element to support life, the European Commission recognised phosphate as a critical raw material in 2014 and placed a greater importance on its sustainable use. Phosphate rock is mined at a rate of 20 million t pa from just a few countries, Morocco, Western Sahara, China, USA and Russia (EC, 2014). The world is therefore reliant on the import of mineral fertilisers resulting in environmental impact as a consequence of mining and transportation. In addition, reliance on a few countries poses a potential threat to food security.

Of the 22 distilleries, 18 had >1 g *L*^−1^ phosphate highlighting the potential for recovery and reuse at a local or national level. Based on the mean value obtained in this study multiplied by the estimated annual production of pot ale and spent wash from whisky distilling in Scotland, this translates to a potential 13,800 t pa of phosphate representing more than half of the annual fertiliser consumed in Scotland.

Over the last few decades there has been an intensive effort to remove phosphate during wastewater treatment to reduce its environmental impact from its discharge to the aquatic environment. This has typically been achieved by chemical precipitation or biological approaches which exploit polyphosphate accumulating microbes. Recently, a transition has been observed in phosphate recovery and reuse, contributing to the development of more sustainable circular economies (Scholz et al., 2013; Dawson and Hilton, 2011). Multiple technologies have been established at scale to recover phosphate from wastewater treatment plants and include precipitation, crystallisation, ion exchange and filtration using membrane technology. These processes typically resulted in the production of magnesium ammonium potassium struvite (MgNH_4_PO_4_ or MgKPO_4_•6H_2_O) or hydroxyapatite (HAP, Ca_5_(PO_4_)_3_OH) and have been well reviewed elsewhere (Ye et al., 2017; Chrispim et al., 2019; Jupp et al., 2021).

Only a few studies have explored removal of phosphate from pot ale (Dionisi et al., 2014; Tokuda et al., 1999). Tokuda et al. (1999) investigated the removal of phosphate using magnesium precipitation to produce struvite and achieve a 90% recovery demonstrating that technologies used in wastewater treatment are also suitable for efficient resource recovery from other feedstocks (Tokuda et al., 1999). Another study by Dionisi et al. (2014) achieved 60% removal of phosphate from pot ale using chitosan adsorption.

Concentrations of other inorganic ions, chloride, nitrate and sulphate were in the milligram per L range. Low and variable concentrations of nitrate (Fig. 4) (10–123 mg *L*^−1^ would be recovered along with the phosphate. Nitrite, which is toxic, was not quantifiable in any pot ale samples complying with Commission Regulation (No. 574/2011) whereby nitrites present in animal feed must not exceed 15 mg kg^−1^.

### 3.5 The metal content of pot ale

Metals for analysis were selected based on their toxicity for future reuse of pot ale, or if sufficient elements were available for future application as a microbial feedstock. The total and soluble metal concentrations found in pot ale was determined (Table 1). Mean total metal concentrations in pot ale were generally <10 mg *L*^−1^ (Cd, Cr, Cu, Fe, Ni, Mn, Pb, Zn) although mean concentrations of Mg were 188 mg *L*^−1^. Most of the Mg was in the soluble fraction (98.5%), whilst the majority of other metals were in the insoluble fraction (Table 1). Magnesium compounds are important in animal feed supplements, fertilisers and for environmental applications such as drinking and waste-water treatment. The high concentrations found in pot ale could be a useful resource, especially if they could be used in-situ to produce the struvite mentioned for phosphate recovery. Although global reserves of magnesium metal and minerals (magnesite and brucite) are estimated to be 12 billion t, it is still considered a critical raw material from an EU perspective. Deposits are primarily in China, hence recovery and reuse at a local level will reduce carbon emissions from production and transportation along with associated environmental impact.

**Table 1:** Mean and range metal concentrations for soluble and total fractions quantified in pot ale.

Pot ale	Cd	Cr	Cu	Fe	Ni	Mg	Mn	Pb	Zn
Soluble metal concentrations (mg *L*^−1^)									
Mean*	n.d.	0.03 ± 0.11	0.53 ± 0.86	0.38 ± 0.50	n.d.	185 ± 38	0.45 ± 1.17	0.06 ± 0.04	2.14 ± 3.70
Range	n.d.	<0.007 – 0.50	<0.01 – 2.97	<0.005 – 1.69	n.d.	90.0 – 234	<0.001 – 4.80	<0.042 – 0.16	<0.006 – 15.9
Total metal concentrations (mg *L*^−1^)									
Mean*	0.02 ± 0.04	1.35 ± 1.89	5.62 ± 5.14	8.65 ± 8.27	1.79 ± 1.29	188 ± 46.1	2.18 ± 1.62	2.93 ± 1.72	4.91 ± 4.97
Range	<0.003 – 0.14	<0.007 – 7.84	0.63 – 18.8	36.7 –2.01	<0.015 – 4.92	126 – 305	<0.001 – 6.33	0.04 – 5.72	0.01 – 18.6
Proportion bound to solids (%)	100	97.8	90.6	95.1	100	1.5	79.4	80.0	56.4

*Values below the limit of quantification (LOQ) were assigned half the LOQ value.

n.d. not determined.

### 3.6 The total organic carbon content of pot ale

**Fig. 5:**
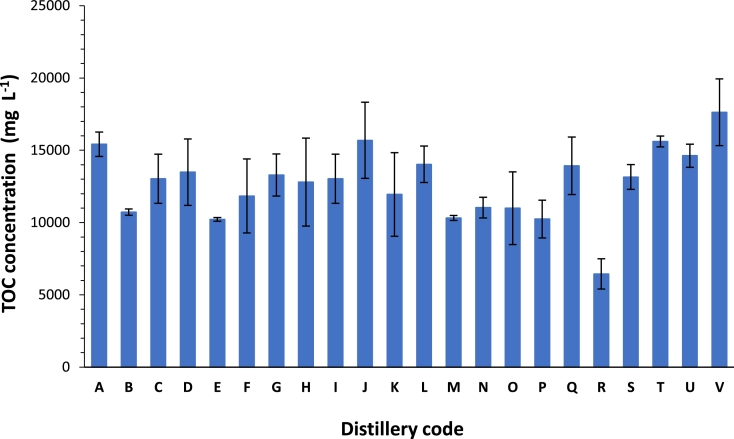
The concentration of total organic carbon (TOC) in pot ale from each distillery. The error bars are representative of the standard deviation from the mean (*n* = 3).

The mean concentration of total organic carbon (TOC) in pot ale was 12.7 g *L*^−1^. With the exception of Distillery R (TOC concentration: 6.5 g *L*^−1^), TOC concentrations ranged from 10.2 g *L*^−1^ to 17.6 g *L*^−1^ (Fig. 5) and were in agreement with values of 16.4 g *L*^−1^ reported by Tokuda et al. (1999). High TOC reflects the presence of proteins, amino acids, organic acids and carbohydrates present in pot ale (Traub-Modinger, 2015). The chemical oxygen demand (COD) is an important factor when considering the chemical properties of TOC present in fermentation co-products, particularly for environmental regulatory purposes. The relationship between the COD and TOC is generally linear, although the precise ratio is dependant on the organic components of the given material (Bernardo et al., 2012). Using existing literature and the TOC data gathered in this study, it can be estimated that the COD/TOC ratio of pot ale is approximately 3:1 (Barrena et al., 2018; Traub-Modinger et al., 2015; Tokuda et al., 1999). With societal drivers to reduce carbon emissions it is becoming more common to make use of fixed carbon. The anaerobic digestion of pot ale along with other distillery co-products to reduce COD and produce biogas is becoming increasingly popular (Barrena et al., 2018; Bustamante et al., 2005; Goodwin et al., 2001; Satayawali and Balakrishnan, 2008). A study by Goodwin et al. (2001) achieved a COD removal rate of between 70 and 90% from diluted pot ale using an up flow anaerobic sludge blanket digester (UASB) at a mean organic loading rate of 5.46 kg COD m^3^ day. Similarly, a study by Tokuda et al. (1999) achieved a COD removal rate of between 70 and 80% for a loading rate of 20 kg COD m^3^ day. While the addition of high protein feedstock to AD systems can lead to the build-up of ammonia that may have an inhibitory effect on methanogenic activity (Ariunbaatar et al., 2015), a study by Barrena et al. (2018) demonstrated no significant difference between un-treated and deproteinated. They investigated the batch anaerobic digestion of deproteinated pot ale from five malt whisky distilleries demonstrating that there was no significant difference in methane yield between treated and un-treated pot ale illustrating that pot ale as a feed stock can be used for multiple co-products.

### 3.7 Total carbohydrate content

The mean total free monosaccharide concentrations were 0.6 g *L*^−1^ and ranged from 0.12 to 1.9 g *L*^−1^ (Table 2), which were low as expected, since distilleries optimise the conversion of starch to maximise alcohol yield. Once hydrolysed, the mean total concentration of monosaccharides was 18.4 g *L*^−1^ with a range of 5.08 to 45.5 g *L*^−1^ highlighting potential valorisation opportunities. Concentrations of individual hydrolysed monosaccharides decreased in the order of glucose > arabinose > xylose > mannose > galactose. Differences were observed in carbohydrate composition from pot ale collected from grain and malt distilleries and were reflective of the differences in grain i.e. wheat versus barley cell wall composition (arabinoxylan polymers and ß-glucan polymers respectively). Pot ale sampled from grain distilleries (R and Q) had far greater mean concentrations of arabinose (1.70 g *L*^−1^), galactose (0.64 g *L*^−1^) and xylose (3.05 g *L*^−1^) when compared to values observed from malt distilleries (arabinose: 0.96 g *L* ^−^ ^1^; galactose: 0.36 g *L*^−1^ and xylose: 1.28 g *L*^−1^) (Table S.2, supplementary information). With the exception of distilleries P, S and V, pot ale collected from malt distilleries generally had greater concentrations of glucose present (mean: 17.4 g *L*^−1^), probably from mixed-linked *β*-glucan, when compared to grain distilleries (mean: 5.65 g *L*^−1^). It would be desirable to have a better representation of data from grain distilleries to confirm this observation, particularly as other cereals such as maize is also used in grain distilleries.

**Table 2:** Mean and range carbohydrate concentrations for free (mg *L*^−1^) and hydrolysed monosaccharides (g *L*^−1^) in pot ale.

Monosaccharides	Arabinose		Galactose		Glucose	Mannose	Rhamnose		Xylose	Total	Glucose based quantification
Free monosaccharides concentrations (mg *L*^−1^)											
Mean*		119 ± 57.1		46.2 ± 27.5	283 ± 329	n.d.	7.58 ± 3.40	115 ± 37.2		581 ± 358	
Range		18.9 – 250		<1.00 – 83.9	11.3 – 688	n.d.	<1.00 – 17.0	41.5 – 175		123 – 1920	
Hydrolysed monosaccharides concentrations (g *L*^−1^)											
Mean*		1.00 ± 0.31		0.39 ± 0.12	14.5 ± 8.82	0.93 ± 0.26	n.d.	1.44 ± 0.63		18.4 ± 8.95	18.8 ± 6.81
Range		0.44 – 2.25		0.15 – 0.72	3.03 – 40.7	0.30 – 1.38	n.d.	0.48 – 3.52		5.08 – 45.5	7.47 – 34.9

* Values below the limit of quantification (LOQ) were assigned half the LOQ value; n.d. not determine.

The high concentration of monosaccharides in pot ale after hydrolysis makes it a potential feedstock to produce second-generation biofuels. Second generation biofuels can be defined as biofuels deriving from food waste or other lignocellulose feedstocks. These feedstocks are preferential to the first-generation biofuels as they do not require arable land and instead use by-products from other production processes. Carbohydrate rich feedstocks that have been utilised to produce second-generation biofuels include cheese whey, potato starch and instant noodle waste (Zhang et al., 2016). Similar, to pot ale these feedstocks generally must be hydrolysed in order to make their carbon sources more bioavailable for fermentation. Instant noodle waste and cheese whey were shown to have 167 g *L*^−1^and 150 g *L*^−1^ of sugars after hydrolysis, respectively (Zhang et al., 2016). The resulting starch was then utilised to produce bio ethanol via fermentation with S. cerevisiae with yields of > 60 g *L*^−1^ (Zhang et al., 2016). In comparison to these feedstocks, pot ale has a lower concentration of potentially fermentable sugars present as they have been used during fermentation.

### 3.8 Impact of distillery process variables on resource composition

**Fig. 6:**
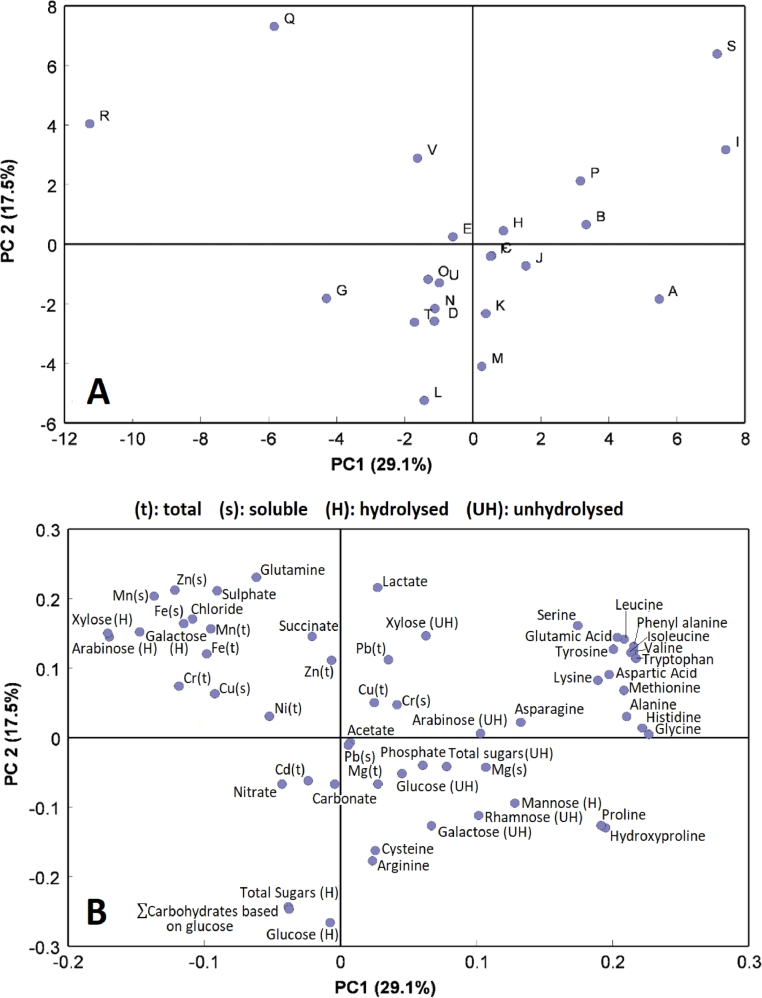
Corresponding scores plot for the distilleries (A) and loadings of the compositional parameters (B) across PCs 1 and 2 of the Principal Component Analysis of the data from all 22 distilleries.

The key parameters (barley variety, malt peating level, wort clarity, yeast strain, length of fermentation) that may influence the chemical composition of pot ale were collected from all distilleries (Table S.1, supplementary information). Despite significant variation in production parameters such as fermentation times ranging from 50 to 114 h, there were only slight differences in composition of soluble pot ale supporting this is a consistent and reliable feedstock for resource recovery/reuse. Principal Component Analysis (PCA) of the compositional data clearly separated the malt distilleries from grain distilleries R & Q (Fig. 6), which accounted for over 29% of the variance in the original data. The grain distillery samples were associated with higher levels of chloride, sulphate, glutamine, and three hydrolysed monosaccharides (xylose, arabinose and galactose), as discussed (Section 3.7). No other trends in data were observed showing a relative consistent composition of pot ale across the malt distilleries.

The twenty malt distilleries were relatively closely clustered on PCs 1 and 2, though Distilleries S and I were seen to be slightly separated from the rest of the group having relatively high levels of several amino acids. Further analysis showed that these had longer fermentation times (Fig S.1, supplementary information).

PCA analysis demonstrated that there was no link between time in transit and pot ale composition confirming the effectiveness of the robust sampling protocol.

## 4 Pot ale/spent wash as a feedstock for microbial production of chemicals and polymers

In addition to extensive resource recovery, pot ale/spent wash could alternatively be used as a feedstock for microbial production of important chemicals and polymers contributing to the transition to net zero. To date there are few examples of this, including, pot ale in combination with draff used for bacterial acetone-butanol-ethanol (ABE) fermentation for the production of biobutanol, acetone, ethanol and animal feed by Celtic Renewables (Scottish Government, 2015). This fermentation approach was used for solvent production until the 1960s, although not using waste feedstocks.

Other studies have demonstrated the use of fungi such as Aspergillus niger for lipid production for 3rd generation biodiesel, when grown on pure vinasse (distillery spent wash) from ethanol distilleries which has some similarity in composition to pot ale (Hoarau et al., 2018). Alternatively, edible filamentous fungi such as Neurospora intermedia and Aspergillus oryzae, have been grown on vinasse producing high yields (>200 g *L*^−1^) of dry proteinaceous biomass suitable for aquaculture/agriculture (Nair and Taherzadeh, 2016).

In addition to fuel and feed, we are reliant on plastics, 98% of which are derived from petroleum which is not sustainable and further compounded by the lack of biodegradation resulting in serious global pollution. In addition to reduced use of plastics there is a need for increased production of bioplastics. Polyhydroxalkanoates (PHAs) are a group of polyesters produced mainly as storage compounds in bacteria and archaea, where biosynthesis is triggered by nutrient limitation and stress. These biopolymers have been used to make high quality plastic with properties very similar to polypropylene, with the added advantage of it being completely biodegradable in 3–9 m. Multiple studies have demonstrated the potential to produce PHAs at up to 70% wet weight on vinasse by Haloferax mediterranei. PHAs and biohydrogen have also been produced by fermentation of Bacillus tequilensis on vinasse spent wash (Amulya et al., 2014). These are a few examples to illustrate potential opportunities paving the way for future exploitation of pot ale and economic growth.

## 5 Resource recovery options

In evaluation of practical, economical and environmentally sustainable options for recovery of these valuable resources in pot ale, the wider distillation process and associated co-products must be considered. Central to this is the inclusion of on-site or local anaerobic digester as a means of using co-product for renewable energy generation and production of high quality, stable fertiliser whilst at the same time elimination transport and disposal costs. AD use is increasing as many distilleries are moving successfully towards use of their co-products, particularly draff and pot ale in the move to net zero by 2040.

However, given the extent of valuable resources identified in the soluble portion of pot ale, a more strategic biorefinery approach must be considered taking into account technologies and cost. Potential options are discussed based on the availability of an AD plant.

**Fig. 7:**
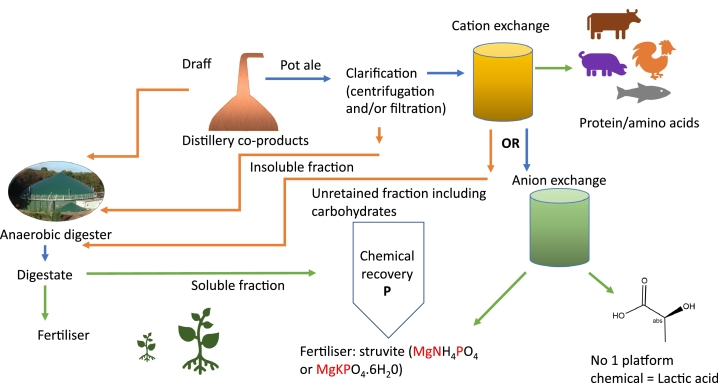
Potential resource recovery in a pot ale biorefinery - green lines shown product recovery streams and brown lines show material flow of remaining biomass as feed for anaerobic digestion.

In addition to pot ale, draff or spent grains (wet grains) are produced and are an excellent feedstock for AD. In our theoretical system (Fig. 7) the draff goes directly to AD whereas the pot ale is separated so the soluble fraction can be easily processed and the separated solids which are mainly dead yeast cells contribute to AD feedstock. This separation could be achieved by centrifugation or filtration to yield a clear soluble fraction that can be efficiently processed.

Since protein recovery from pot ale using a patented ion exchange process has been successful in large scale trials demonstrating that even focus on a single product, a nutritionally excellent protein for aquaculture and animal feed, can be economically and environmentally sustainable. Pot ale would need to be clarified by centrifugation or filtration allowing the insoluble fraction to contribute to AD feed whilst the soluble fraction would be applied to industrial scale cation exchange column for protein purification, this would be the first product reclaimed allowing the unretained material rich in carbohydrates to serve as AD feedstock. Removal of protein from pot ale prior to AD has been shown to have no impact on AD performance (Barrena et al., 2018).

Given the importance of phosphate recovery and extensive research and commercialisation of recovery from both solid and liquid fractions following wastewater treatment, the process described here would take the soluble waste from the AD process to recover phosphate. Chemical precipitation methods are relatively simple, give high recovery of crystals that can be readily dewatered, on the downside large quantities of chemicals are needed and waste sludges produced.

Struvite (magnesium-ammonium-phosphate (MAP) MgNH_4_PO_4_) production, giving a slow-release fertiliser is an appealing route for resource recovery using proven technology at scale, with multiple commercial operators across the world (Desmidt et al., 2015; Ye et al., 2017). Most commonly, oxides or salts of magnesium (MgO, Mg(OH)_2_, MgCl_2_) are used to drive precipitation at pH-8–9. Since pot ale has a typical pH 3.5–4, extensive pH control would be needed to achieve the necessary alkaline pH to facilitate precipitation. This could be achieved by dosing NaOH, however one approach to avoiding this is the use of aeration to drive up the pH as well as provide good mixing with Mg salt as in the AirPrex® process using a continuous stirred tank reactors resulting in 80–90% P recovery without NaOH. It is quite possible pot ale may need a combination of NaOH dosing and aeration to obtain a suitable pH for struvite precipitation. In addition, aeration by CO_2_ stripping may be considered as a negative strategy although such a process must be evaluated economically and environmentally prior to implementation.

Most work on P recovery for struvite production has been from wastewater where the concentration of P is <10 mg/L. and the volumes are huge, in contrast to pot ale with higher concentrations and lower volumes. Mg costs for struvite production in distilleries in coastal locations could be reduced by the use of seawater which contains a high concentration of Mg^2+^ ions (Kumar and Pal, 2015). In addition, pot ale has been shown to contain high concentrations of K, approx. 1 g/L. which may be used to drive the production of potassium struvite (MgKPO_4_.6H_2_0). Clearly further research on simple chemical precipitation as a practical option for P recovery from pot ale is required although it has been shown to be feasible at pilot scale (Tokuda et al., 1999). Cost of struvite recovery from wastewater ranges from $0.82–0.95 /kg•PTotal with a market prices $2–3/kg•PTotal twice that of raw phosphate rock. Market prices need to change to reflect carbon footprint, or governments need to incentivise industry on using more expensive but sustainable products. In addition, active recovery of P will protect excess transit into watersheds which result in eutrophication with many associated issues.

In order to maximise the resource reclamation from the pot ale, an approach using ion exchange would facilitate recovery of lactic acid and possibly succinic acid as well as phosphate. Traditional lactic acid is recovered from fermentation broths using chemical precipitation, using large amounts of chemicals and with many filtration steps, but as demand is increasing for this compound, many alternative methods have been explored and applied (de Oliveira et al., 2018). The logical process in the pot ale biorefinery would take the unretained flow stream from the cation exchanger and use weak base anion exchangers such as Amberlite-IRA 67, to make best use of the low pH of the pot ale, typically lower than the pK_a_ of lactic acid (3.86) for production of high quality lactic acid. In addition, phosphate could also be recovered at this step for production of struvite. This approach would use less chemicals and energy with the advantage that the ion exchange columns can be reused many times.

## 6 Conclusions

The Scotch Whisky Industry continues to make excellent progress towards being carbon neutral and provides a benchmark for other industries. This study highlights the potential for resource recovery from the soluble portion of whisky pot ale and spent wash in Scotland contributing to a more sustainable and circular economy. Beyond this study, these resources are also available from spent wash from other distilled spirits including bioethanol representing a huge global resource. As well as recovery, these may be reused as a feedstock for microbiological production of lipids, protein and polymers as alternatives to those currently obtained from petroleum-based processes. Ultimately, the outcome will be driven by local needs and government/global targets such as zero carbon by 2050. However, with increased implementation of AD at many distillery sites, the most feasible approach in the short term would be to recover key critical materials such as protein & amino acids for feed, lactic acid as a platform chemical along with phosphate, nitrate, magnesium, potassium for fertiliser with remaining material recycled to AD. It is essential to recognise the valuable resources we have on our doorstep, such as pot ale, and transition to their use in sustainable applications in a circular bioeconomy.

## CRediT authorship contribution statement

**Christine Edwards:** Conceptualization, Writing – original draft, Resources, Supervision, Funding acquisition. **Calum C. McNerney:** Conceptualization, Investigation, Methodology, Data curation, Writing – review & editing. **Linda A. Lawton:** Conceptualization, Resources, Supervision, Writing – review & editing. **Joseph Palmer:** Methodology, Data curation, Formal analysis, Writing – review & editing. **Kenneth Macgregor:** Methodology, Data curation, Formal analysis, Writing – review & editing. **Frances Jack:** Resources, Writing – review & editing. **Peter Cockburn:** Methodology, Formal analysis. **Amy Plummer:** Methodology, Data curation. **Alison Lovegrove:** Methodology, Data curation, Formal analysis, Writing – review & editing, Funding acquisition. **Abigail Wood:** Methodology, Formal analysis.

## Declaration of Competing Interest

Dear Editor,

I would like to submit the accompanying manuscript for consideration of publication in Resources, Conservation & Recycling.

RE: Recoverable Resources from Pot Ale & Spent Wash from Scotch Whisky Production

I would like to confirm that there are no conflicts of interest associated with this manuscript.

## Appendix Supplementary materials

Image, application 1
