# Supplementary material for: Recoverable resources from pot ale & spent wash from Scotch Whisky production
Source: Resour Conserv Recycl. 2022 Apr;179:106114. doi: 10.1016/j.resconrec.2021.106114 (PMC8803549; doi:10.1016/j.resconrec.2021.106114)
Supplement: Supplementary file 1 [file mmc1.docx]

**Supplementary information**

Recoverable Resources from Pot Ale & Spent Wash from Scotch Whisky Production

Calum C. McNerney ^a^, Linda A. Lawton^a^, Joseph Palmer^a^, Kenneth Macgregor ^b^, Frances Jack ^b,^ Peter Cockburn ^c^, Amy Plummer ^d^, Alison Lovegrove ^d^, Abigail Wood ^d^, Christine Edwards ^a*^

a. The School of Pharmacy and Life Science, Robert Gordon University, Aberdeen, AB10 7GJ, United Kingdom.

b. The Scotch Whisky Research Institute, The Robertson Trust Building, Research Avenue North, Riccarton, Edinburgh, EH14 4AP, United Kingdom

c. Diageo, International Technical Centre, Glenochil, Menstrie, Clackmannanshire, Scotland, FK11 7ES

d. Rothamsted Research, West Common, Harpenden, Hertfordshire, AL5 2JQ, United Kingdom

*****Corresponding author: Christine Edwards. Email: [c.edwards@rgu.ac.uk](mailto:c.edwards@rgu.ac.uk)

| **Distillery code** | **Barley**  **variety** | **Malt peating level** | **Wort clarity** | **Yeast**  **strain** | **Length of fermentation (hours)** | **Time in transit (days)** |
| --- | --- | --- | --- | --- | --- | --- |
| **A** | Sienna | unpeated | clear | MG + | 62 | 6 |
| **B** | Concerto | unpeated | clear | Pinnacle | 70 | 6 |
| **C** | Concerto | unpeated | cloudy | Pinnacle | 50 | 3 |
| **D** | Concerto | unpeated | cloudy | Kerry | 70 | 6 |
| **E** | Concerto | unpeated | clear | Pinnacle | 60 | 6 |
| **F** | Concerto | unpeated | clear | MG+ | 56 | 4 |
| **G** | Concerto | unpeated | cloudy | Kerry | 85 | 5 |
| **H** | Concerto | heavily peated | clear | Pinnacle | 53 | 4 |
| **I** | Concerto | heavily peated | clear | Unknown | 109 | 6 |
| **J** | Concerto | heavily peated | clear | Pinnacle | 59 | 6 |
| **K** | Laureate | unpeated | clear | Kerry | 60 | 4 |
| **L** | Concerto | unpeated | unknown | Kerry | 60 | 6 |
| **M** | Concerto | unpeated | clear | MG+ | 56 | 8 |
| **N** | concerto | unpeated | unknown | Pinnacle | 48 | 5 |
| **O** | Concerto | unpeated | clear | Pinnacle | 51 | 1 |
| **P** | Concerto | unpeated | clear | Pinnacle | 65 | 6 |
| **Q** | Wheat |  |  | Kerry | 57 | 6 |
| **R** | Wheat |  |  | Kerry | 91 | 8 |
| **S** | Concerto | unpeated | clear | Kerry | 114 | 2 |
| **T** | Concerto | unpeated | clear | MG + | 97, 54, 52 | 5 |
| **U** | Concerto | medium peated | unknown | Kerry | Unknown | 4 |
| **V** | Concerto | medium peated | unknown | Kerry M | 98 | 6 |

**Table S.1** Summary of a Scotland wide pot ale and spent wash survey

**Table S.2:** Free (mg L^−1^ ), hydrolysed (g L^−1^ ), and glucose-based (g L^−1^ ) carbohydrate concentrations observed in pot ale collected from 22 Scotch Whisky distilleries**.**

|  | **A** | **B** | **C** | **D** | **E** | **F** | **G** | **H** | **I** | **J** | **K** | **L** | **M** | **N** | **O** | **P** | **Q** | **R** | **S** | **T** | **U** | **V** |
| --- | --- | --- | --- | --- | --- | --- | --- | --- | --- | --- | --- | --- | --- | --- | --- | --- | --- | --- | --- | --- | --- | --- |
|  |  | | | | | | | | | | | | | | | | | | | | | |
| ***Free carbohydrates*** | |  | **(in mg L^−1^ )** | |  |  |  |  |  |  |  |  |  |  |  |  |  |  |  |  |  |  |
| **Arabinose** | 143 | 56 | 33 | 185 | 116 | 117 | 62 | 77 | 184 | 48 | 104 | 163 | 142 | 141 | 136 | 250 | 63 | 19 | 165 | 129 | 164 | 128 |
| **Galactose** | 84 | 47 | 21 | 32 | <1 | 59 | <1 | 50 | 49 | 66 | 59 | 78 | 62 | 62 | 56 | 74 | 9 | 28 | 27 | 72 | 83 | <1 |
| **Glucose** | 232 | 688 | 524 | 1580 | 166 | 166 | 11 | 192 | 245 | 152 | 155 | 292 | 114 | 265 | 154 | 257 | 34 | 35 | 233 | 103 | 433 | 195 |
| **Rhamnose** | 17 | 8 | 11 | 11 | 3 | 10 | 4 | 7 | 6 | 9 | 7 | 8 | 10 | 7 | 8 | 5 | 10 | <1 | 5 | 8 | 8 | 4 |
| **Xylose** | 165 | 86 | 125 | 110 | 84 | 125 | 60 | 102 | 115 | 84 | 115 | 62 | 118 | 120 | 132 | 175 | 195 | 42 | 143 | 122 | 141 | 109 |
| **Total** | 641 | 884 | 714 | 1920 | 369 | 477 | 138 | 429 | 599 | 358 | 439 | 603 | 446 | 595 | 486 | 761 | 311 | 123 | 573 | 434 | 799 | 437 |
| ***Hydrolysed carbohydrates*** | | | **(in g L^−1^ )** | |  |  |  |  |  |  |  |  |  |  |  |  |  |  |  |  |  |  |
| **Arabinose** | 1.15 | 1.09 | 0.99 | 0.89 | 0.63 | 1.13 | 0.83 | 0.77 | 1.01 | 0.91 | 0.78 | 1.25 | 1.06 | 1.07 | 1.04 | 0.44 | 1.95 | 1.44 | 0.84 | 1.14 | 0.84 | 0.69 |
| **Galactose** | 0.58 | 0.41 | 0.34 | 0.41 | 0.15 | 0.41 | 0.25 | 0.30 | 0.38 | 0.33 | 0.36 | 0.47 | 0.39 | 0.38 | 0.37 | 0.26 | 0.72 | 0.55 | 0.36 | 0.44 | 0.39 | 0.26 |
| **Glucose** | 23.2 | 15.2 | 20.7 | 17.2 | 6.03 | 15.4 | 13.2 | 12.2 | 9.41 | 16.3 | 13.9 | 40.7 | 26.1 | 16.8 | 16.2 | 3.03 | 5.62 | 5.69 | 5.81 | 17.0 | 16.7 | 3.45 |
| **Mannose** | 1.38 | 1.04 | 0.94 | 1.10 | 0.96 | 1.31 | 0.92 | 0.79 | 1.24 | 0.88 | 0.93 | 1.79 | 1.31 | 1.12 | 0.96 | 0.87 | 0.56 | 0.30 | 1.10 | 1.02 | 0.86 | 0.68 |
| **Xylose** | 1.45 | 1.52 | 1.52 | 1.15 | 0.86 | 1.71 | 1.2 | 1.07 | 1.41 | 1.41 | 1.05 | 1.27 | 1.52 | 1.49 | 1.34 | 0.48 | 3.52 | 2.57 | 1.09 | 1.49 | 1.09 | 0.87 |
| **Total** | 27.7 | 19.3 | 24.5 | 20.8 | 8.63 | 20.0 | 16.4 | 15.2 | 13.5 | 19.8 | 17.1 | 45.5 | 30.4 | 20.9 | 19.9 | 5.08 | 12.4 | 10.6 | 9.19 | 21.1 | 19.9 | 5.95 |
| **Sum total** | 28.3 | 20.2 | 25.2 | 22.7 | 9.00 | 20.5 | 16.5 | 15.6 | 14.1 | 20.2 | 17.5 | 46.1 | 30.8 | 21.5 | 20.4 | 5.84 | 12.7 | 10.7 | 9.76 | 21.5 | 20.7 | 6.39 |
| **Total Carbohydrate based on Glucose response** | 29.3 | 19.2 | 22.5 | 21.5 | 9.81 | 18.1 | 16.0 | 17.1 | 14.8 | 19.7 | 20.0 | 40.5 | 26.1 | 16.8 | 16.2 | 3.03 | 6.33 | 5.69 | 5.81 | 17.0 | 20.7 | 3.45 |

**Table S.3:** Mean concentration of free amino acids mg L^−1^ (highlighted amino acids are essential in feed) in each distillery sample. N = 3

|  | **A** | **B** | **C** | **D** | **E** | **F** | **G** | **H** | **I** | **J** | **K** | **L** | **M** | **N** | **O** | **P** | **Q** | **R** | **S** | **T** | **U** | **V** |
| --- | --- | --- | --- | --- | --- | --- | --- | --- | --- | --- | --- | --- | --- | --- | --- | --- | --- | --- | --- | --- | --- | --- |
|  |  | | | | | | | | | | | | | | | | | | | | | |
| **Alanine** | 229 | 212 | 164 | 103 | 113 | 168 | 105 | 230 | 271 | 249 | 180 | 158 | 119 | 99 | 103 | 177 | 96 | 32 | 247 | 114 | 171 | 119 |
| **Arginine** | 116 | 13 | 14 | 33 | 18 | 15 | 14 | 13 | 13 | 15 | 102 | 88 | 93 | 23 | 59 | 13 | 9 | 9 | 13 | 71 | 21 | 62 |
| **Asparagine** | 10 | 42 | 20 | 7 | 7 | 6 | 6 | 31 | 49 | 39 | 25 | 24 | 6 | 3 | 4 | 10 | 7 | 5 | 24 | 21 | 21 | 5 |
| **Aspartic acid** | 103 | 63 | 65 | 39 | 50 | 30 | 4 | 22 | 106 | 41 | 34 | 22 | 56 | 46 | 53 | 54 | 16 | 4 | 132 | 20 | 27 | 68 |
| **Cysteine** | 60 | 1 | 2 | 2 | 1 | 2 | 1 | 2 | 2 | 2 | 85 | 51 | 86 | 1 | 1 | 2 | 1 | 4 | 3 | 30 | 2 | 1 |
| **Glutamic acid** | 101 | 81 | 62 | 75 | 58 | 89 | 33 | 92 | 142 | 98 | 66 | 113 | 64 | 57 | 59 | 110 | 113 | 46 | 183 | 87 | 83 | 75 |
| **Glutamine** | 2 | 3 | 1 | 1 | 1 | 0 | 0 | 0 | 3 | 1 | 1 | 1 | 1 | 0 | 0 | 2 | 6 | 8 | 4 | 1 | 0 | 0 |
| **Glycine** | 136 | 113 | 95 | 69 | 82 | 106 | 65 | 120 | 157 | 141 | 89 | 83 | 83 | 76 | 75 | 109 | 38 | 3 | 168 | 102 | 108 | 73 |
| **Histidine** | 92 | 58 | 43 | 43 | 41 | 45 | 34 | 65 | 83 | 75 | 41 | 46 | 46 | 38 | 37 | 55 | 17 | 3 | 101 | 45 | 58 | 42 |
| **Hydroxyproline** | 2 | 2 | 1 | 1 | 1 | 1 | 1 | 1 | 2 | 1 | 1 | 2 | 2 | 1 | 1 | 1 | 1 | 0 | 1 | 1 | 2 | 1 |
| **Isoleucine** | 53 | 50 | 40 | 15 | 28 | 42 | 9 | 38 | 82 | 31 | 29 | 13 | 30 | 25 | 24 | 47 | 26 | 2 | 93 | 16 | 16 | 25 |
| **Leucine** | 105 | 105 | 77 | 31 | 54 | 82 | 14 | 77 | 138 | 62 | 63 | 19 | 59 | 49 | 45 | 85 | 74 | 4 | 140 | 26 | 37 | 48 |
| **Lysine** | 120 | 203 | 116 | 75 | 103 | 34 | 42 | 26 | 241 | 119 | 59 | 46 | 64 | 109 | 89 | 122 | 26 | 11 | 195 | 52 | 93 | 102 |
| **Methionine** | 55 | 63 | 47 | 22 | 33 | 41 | 9 | 30 | 50 | 31 | 25 | 16 | 39 | 29 | 23 | 41 | 18 | 1 | 71 | 13 | 16 | 18 |
| **Phenylalanine** | 88 | 79 | 61 | 29 | 45 | 61 | 18 | 63 | 105 | 52 | 52 | 25 | 55 | 41 | 37 | 70 | 55 | 2 | 112 | 27 | 36 | 37 |
| **Proline** | 1220 | 771 | 554 | 696 | 629 | 611 | 527 | 759 | 886 | 832 | 669 | 727 | 893 | 663 | 734 | 764 | 288 | 45 | 692 | 671 | 715 | 791 |
| **Serine** | 41 | 41 | 32 | 9 | 16 | 32 | 0 | 27 | 64 | 29 | 21 | 10 | 19 | 11 | 11 | 18 | 38 | 5 | 54 | 13 | 13 | 16 |
| **Threonine** | 62 | 56 | 38 | 20 | 29 | 42 | 7 | 35 | 84 | 42 | 35 | 21 | 33 | 27 | 27 | 59 | 21 | 3 | 108 | 20 | 26 | 30 |
| **Tryptophan** | 21 | 16 | 14 | 5 | 9 | 11 | 2 | 14 | 24 | 12 | 11 | **7** | 9 | 8 | 7 | 14 | 9 | 1 | 28 | 6 | 8 | 7 |
| **Tyrosine** | 40 | 27 | 20 | 10 | 14 | 21 | 6 | 24 | 63 | 24 | 32 | 12 | 17 | 12 | 17 | 29 | 23 | 1 | 50 | 18 | 21 | 24 |
| **Valine** | 85 | 79 | 60 | 26 | 43 | 66 | 18 | 63 | 122 | 62 | 50 | 24 | 49 | 40 | 39 | 76 | 43 | 4 | 134 | 29 | 33 | 42 |
| **Total free amino acids** | 2737 | 2077 | 1525 | 1309 | 1375 | 1505 | 914 | 1731 | 2684 | 1956 | 1669 | 1504 | 1819 | 1356 | 1446 | 1860 | 923 | 195 | 2553 | 1381 | 1505 | 1588 |


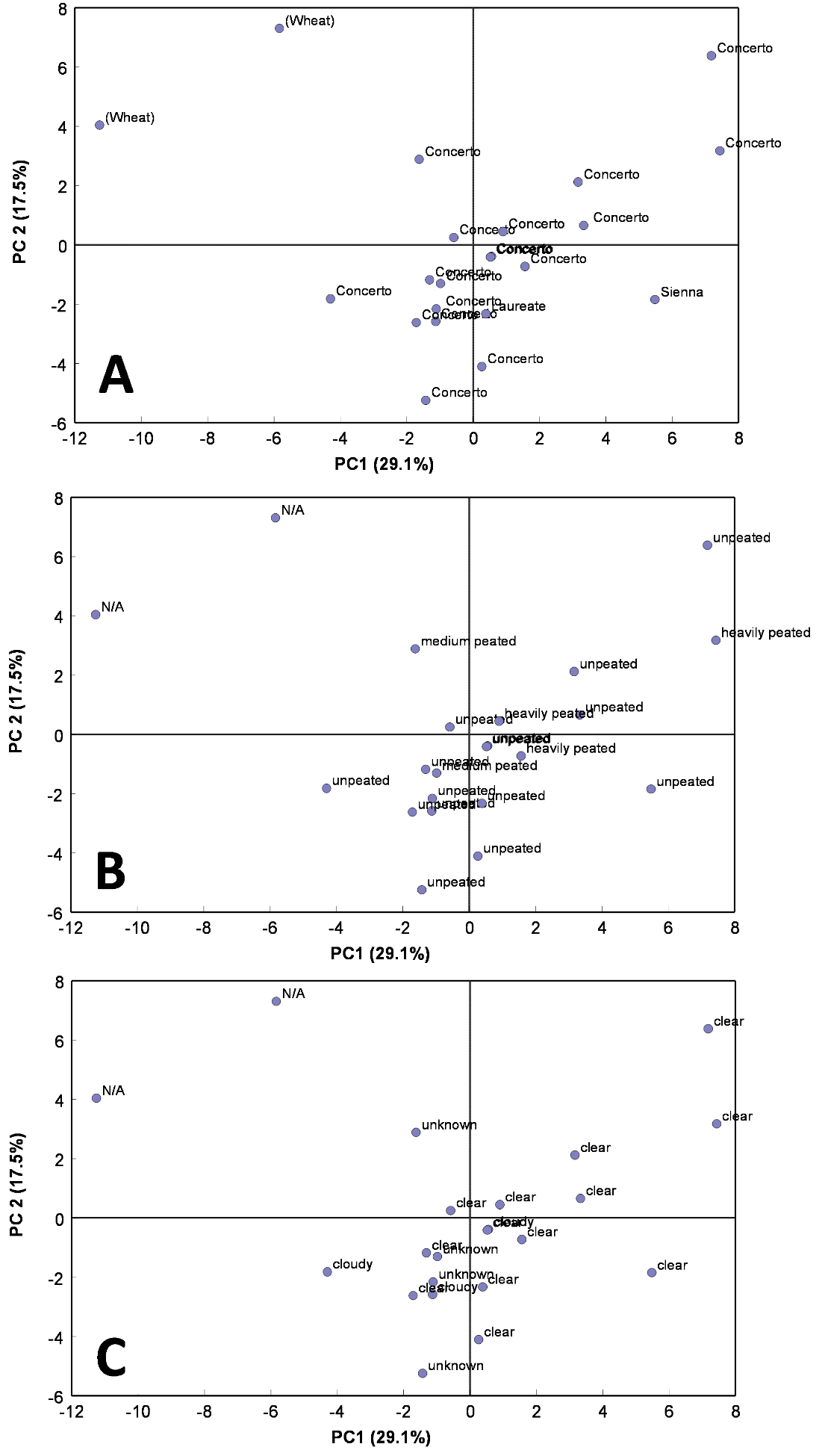
**Figure S.1:** Scores plot for the distilleries across PCs and 2 from the Principle Component Analysis of the data from all 22 distilleries, recoded to show distribution in relation to process parameters: A – barley variety or grain, B – malt peating level and C – wort clarity.

**
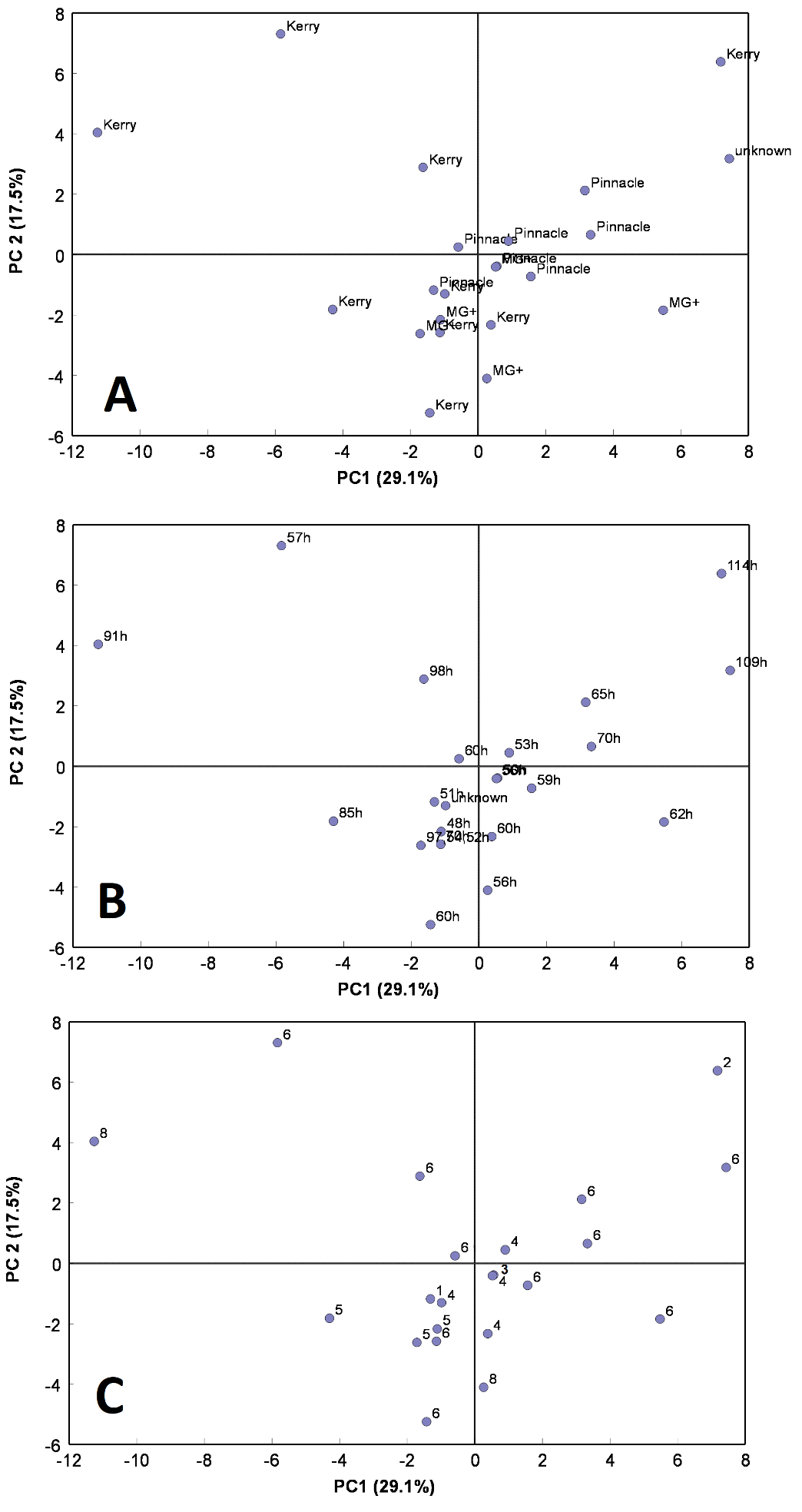
Figure S.2:** Scores plot for the distilleries across PCs and 2 from the Principal Components Analysis of the data from all 22 distilleries, recoded to show distribution in relation to process parameters: A – yeast strain, B – fermentation time and C – time in transit.
